# Protocol: optimised electrophyiological analysis of intact guard cells from *Arabidopsis*

**DOI:** 10.1186/1746-4811-8-15

**Published:** 2012-05-06

**Authors:** Zhong-Hua Chen, Cornelia Eisenach, Xin-Qin Xu, Adrian Hills, Michael R Blatt

**Affiliations:** 1 Laboratory of Plant Physiology and Biophysics, Institute of Molecular, Cell and Systems Biology, University of Glasgow, Glasgow, G12 8QQ, UK; 2 School of Science and Health, University of Western Sydney, Richmond, NSW 2753, Australia

**Keywords:** Microelectrode, K^+^ channel (voltage-gated), Cl^-^ channel, Voltage-gated, Membrane conductance, Mutant analysis, Arabidopsis

## Abstract

Genetic resources available for *Arabidopsis thaliana* make this species particularly attractive as a model for molecular genetic studies of guard cell homeostasis, transport and signalling, but this facility is not matched by accessible tools for quantitative analysis of transport in the intact cell. We have developed a reliable set of procedures for voltage clamp analysis of guard cells from *Arabidopsis* leaves. These procedures greatly simplify electrophysiological recordings, extending the duration of measurements and scope for analysis of the predominant K^+^ and anion channels of intact stomatal guard cells to that achieved previously in work with *Vicia* and tobacco guard cells.

## Introduction

Stomata are pores, commonly found in the epidermis of leaves, and are surrounded by a pair of specialised cells known as guard cells. Guard cells regulate the size of the stomatal pore to balance the exchange CO_2_ for photosynthesis with the need to conserve water [1]. The acquisition of stomata and the leaf cuticle are considered to be key elements in the evolution of advanced terrestrial plants [2] allowing plants to inhabit different and often fluctuating environments while controlling water content. Stomatal pores typically occupy less than 5% of the leaf surface, but they provide for over 90% of the CO_2_ entering the leaf and over 70% of water loss from the plant as a whole [3]. Guard cells respond to a number of well-defined signals – including hormones, light and atmospheric CO_2_ concentration – integrating these signals to regulate stomatal aperture [4,5].

In the past few decades, the combination of physiological and molecular biological methods in the model plant *Arabidopsis thaliana* has greatly advanced our understanding of stomata [1,4-7]. Among these, voltage clamp methods have proven powerful in connecting the molecular and physiological frameworks in an understanding of stomatal function. The voltage clamp itself lies at the core of a toolchest of techniques and provides the essential utility to bring the driving force of membrane voltage under experimental control. By so doing, it enables the dissection, identification and monitoring of ionic currents carried by individual ion transporters – ATP-dependent pumps, ion-coupled carriers and ion channels – across biological membranes [8]. Classic voltage clamp methods rely on impalements with two microelectrodes (or a single microelectrode with two separate barrels) that are used to measure membrane voltage and to pass current for voltage clamping, respectively [8,9]. Because a defined spatial geometry is essential for quantifying current spread under clamp conditions [8-10], these methods have proven highly successful for work primarily on a small number of single-celled species as well as cell types that are easily isolated from their surrounding tissues [11-17].

Since its wider introduction in the 1980's [18,19], the patch clamp variant of the voltage clamp has been widely used in studies of plant ion channels [8,20]. The patch clamp offers a number of advantages for work on plant cells, the most important being the facility for electrical recordings from single cells isolated from almost any surrounding tissue, thereby avoiding the difficulties of electrical coupling via plasmodesmata between cells in situ [21]. It also presents some difficulties. For patch clamp recordings from plant cells it is essential to remove the cell wall, commonly by enzymatic digestion, and to stabilise the protoplast against osmotic swelling in the absence of turgor. Both manipulations affect the underlying homeostatic properties of the cells and must influence their physiological behaviour [22,23]. Additionally, obtaining electrically and mechanically robust seals between the patch electrode and protoplast, and retaining stable measurements without significant “rundown” of currents over long periods of time are often challenging [20,24].

By contrast with many plant cell types [but see Chen et al. [15]], guard cells at maturity do not retain electrical connections with their neighbours [11,25]. They are easily separated by mechanical peeling of leaves [1] and recovered intact with their cell wall within the monolayer of epidermal cells. These features greatly simplify their handling for voltage clamp recordings and analysis, avoiding the need to isolate protoplasts and the technical challenges of the patch clamp. Despite the obvious advantages, only a very few studies [26-28] have made use of microelectrode impalements and classic voltage clamp methods with intact *Arabidopsis* guard cells. A major difficulty in this case has been to obtain reliable measurements over 20–30 min or more, time periods long enough for physiological and pharmacological studies with single cells. Thus, many researchers have relied on statistical approaches in patch recordings from populations of guard cell protoplasts, often without an internal reference for comparisons; simply put, impalement methods have not offered significant benefits in overcoming the problem of ‘rundown’ in channel activities common to patch clamp recording [20,24].

We have revisited the problems of voltage clamp recording from intact *Arabidopsis* guard cells and offer here a few simple procedures that enable classic, two-electrode voltage clamp recordings. Included with this protocol are summaries of results demonstrating its utility in characterising the major ion channel currents and their stability over time periods of one hour or more. The impalement approach greatly simplifies experimental access to these currents and enables physiological studies to be carried out on a cell-by-cell basis.

## Materials

### Plant materials

*Arabidopsis thaliana*. For purposes of demonstration, we included with wild-type (Col0) the nitrate reductase-null mutant *nia1-1/nia2-5* (*nia1nia2*) [29], the ABA-receptor quadruple mutant *pyr1/pyl1/pyl2/pyl4* (*QC3*) [30], the vesicle-trafficking mutant *syp121* (*=syr1*/*pen1*) and its complementation with *SYP121*[31,32], the dehydroascorbate reductase mutant *dhar1-3*[33], and the K^+^ channel mutant *kc1-2*[31].

### Reagents

KCl, Ca(OH)_2_, NaOH, HCl, CsCl, tetraethylammonium chloride (TEA-Cl), potassium acetate (K^+^-Ac), and 2-(N-morpholino)ethanesulfonic acid (MES) analytical grade.

Opening Buffer (OB) for pretreating the stomatal guard cells, comprising 50 mM KCl and 10 mM MES, titrated to its pH 6.1 with NaOH, without added Ca^2+^.

Recording Buffer 1 (RB1) for voltage clamp measurements of K^+^ channel currents, comprising 10 mM KCl and 5 mM MES, titrated to pH 6.1 with Ca(OH)_2_ ([Ca^2+^] = 1 mM).

Recording Buffer 2 (RB2) for voltage clamp measurements of the Cl^-^/anion channel currents, comprising 15 mM TEA-Cl, 15 mM CsCl and 5 mM MES, titrated to pH 6.1 with Ca(OH)_2_([Ca^2+^] = 1 mM).

### Equipment

Environment-controlled growth room

Refrigerator for stratifying seeds at 4°C

Narashige PD5 multi-purpose microelectrode puller or equivalent, modified for multibarrelled microelectrodes [9].

High-impedance (>10^11^ Ω), multi-channel voltage clamp amplifiers and probes [8,9]

Desktop computer and data acquisition system [8,9]

Light microscope with a total magnification at least 400× or higher

12-volt battery for DC power to supply microscope

Huxley-type micromanipulator with carrier (see below) incorporating light-weight micropositioner (e.g. Narishige C2-type micromanipulator)

Faraday cage

Anti-vibration table

Gravity-feed system for switching between experimental solutions [9]

Optically clear and pressure-sensitive silicone adhesive [8,9,12]

Fine-tipped forceps, dressing forceps and razor blades

Glass capillaries for double-barrelled microelectrodes [9]

Two-ml polypropylene pipettes, silicon rubber and 0.5-mm diameter Ag wire for halfcells (see [9] and below)

## Protocol

### Key steps for growing Arabidopsis plants and selecting guard cells for voltage clamp

Growth history has an appreciable impact on stable voltage clamp recordings in *Arabidopsis* guard cells.

**Figure 1:**
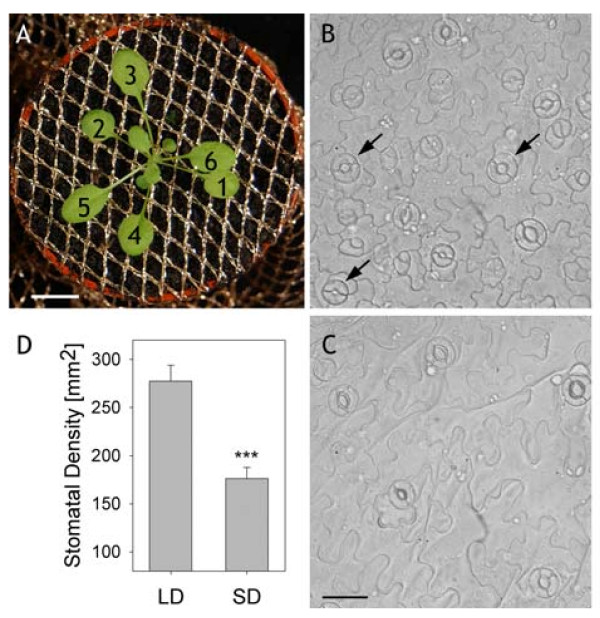
**Growth and selection of *Arabidopsis *guard cells on epidermal peels.** (**A**) Rosette of a plant after 19-d growth at the stage from which epidermal peels were taken for impalements. Plants were grown in individual flower pots, covered with a polyester mesh. True leaves are numbered in order of their appearance. Scale bar, 1 cm. (**B**, **C**) Epidermal peels taken from plants grown under long- and short-day periods, respectively. Note the higher density of stomata and the smaller size of the epidermal cells in (B). Scale bars, 30 μm. Arrows in (B) indicate examples of guard cell pairs favoured for impalement (**D**) Stomatal densities of plants grown under long-day (LD) and short-day (SD) (n = 46). The significance level is indicated with asterisks (P < 0.01).

1. Pretreat compost with Intercept 70WG (Scotts, Ipswich, UK), a systemic insecticide.

2. Sow seeds onto the nutrient-rich Levington F2 + S 3 compost (Coulders, Glasgow, UK) in 60 mm pots covered with polyester mesh (Remnant Kings, Glasgow, UK Figure 1A) to avoid soil contact of the abaxial leaf surface and soil-borne stress factors.

3. Stratify seeds at 4°C, once sown, for 48 hours and leave the seed to germinate under a plastic lid (>95% RH) for one week.

4. Cultivate plants in a controlled environment growth room under long day conditions with 100 μmol m^-2^ s^-1^ light and a light/dark cycle of 16 h/8 h, 22/18°C, and 55/70% RH. Evenly and regularly water plants from below.

5. Transfer pots after one week to propagators. We use propagators with NITEX mesh fabric (mesh opening 200 μm diameter; Sefar, Heiden, Switzerland) over the sides of the covers to permit free air exchange while keeping out insects.

6. In preparation for experiments excise either the 5^th^ or 6^th^ true leaf of three-week-old plants; these leaves display an elliptical shape and are more serrated compared to the older leaves. **NOTE**: *There is a correlation between stomatal responsiveness and stomatal age, the most responsive stomata often occur on leaves with higher stomatal densities, many stomatal primordia and smaller epidermal cells (Figure*1*B and C). Successful impalements yield similar currents under voltage clamp when recorded from guard cells of plants grown under long- and short-day conditions. Nonetheless, we favour plants grown under long days, as growth under short days gives lower stomatal densities (Figure*1*D).*

7. Pretreat the glass of the measuring chamber, coating it with Dow-Corning silicon prosthetic adhesive (Factor II, Tucson, USA; see [9]). **NOTE**: *Silicon adhesive is pressure-sensitive and optically clear. Once dried, it remains useable for many weeks, even under water. However, the solvent used in the adhesive must evaporate before use or it will kill the cells.*

8. Excise the epidermis of the leaf by wrapping the leaf over a finger, adaxial side down, cut into the mesophyll near the base of the mid-vein with forceps, and lift the abaxial epidermis away from the mid vein towards the leave margin. Gently replace peel against the mesophyll, keeping a gentle tension to avoid folds, then cut at the end of the peel near the leaf margin using a fresh (sharp) razor blade. **NOTE**: *It is often easier to peel away the epidermis some minutes after excision when the leaf is less turgid, and to work from the petiole to the apex of the leaf. Ideally, epidermal peels should be free from wrinkles, folds, dirt and, once mounted, air bubbles. Successful impalements are best obtained from open stomata with young guard cells (arrows, Figure*1*B), as judged by the thickness of the stomatal lip and squat shape of the guard cells.*

9. Press the abaxial side of the leaf with the excised epidermal peel gently onto the prosthetic adhesive coating of the measuring chamber glass. Remove the remaining leaf tissue and cover the epidermal peel immediately with OB to prevent it drying.

### Key steps for pulling microelectrodes

The volume of an *Arabidopsis* guard cell is typically 10-15% that of *Vicia* and tobacco guard cells. Thus, microelectrodes with input resistances near 100 MΩ when filled with 200 mM K^+^-Ac, such as have been used in the past [13,34], are not suitable and generally give a low rate of success and a high leak conductance with little evidence of selective transport activity.

**Figure 2:**
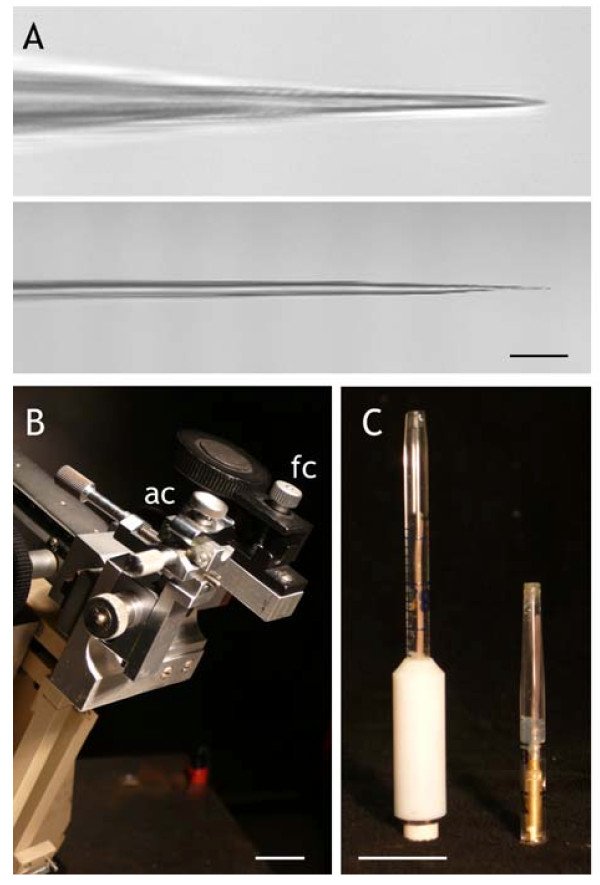
**Mechanical improvements for *Arabidopsis *guard cell impalement. ** (**A**) Double-barrelled microelectrodes pulled with settings for *Vicia* (*above*) and for *Arabidopsis *(*below*), in the latter case showing a 1–1.5^o^ taper to the final 10 μm of the tip. The extreme tips of both microelectrodes are below the resolution of the light microscope. Scale bars, 10 μm. (**B**) A custom-built brace with a fixed clamp (fc) for one amplifier headstage and a second, adjustable clamp (ac) provided by a Narashige C2 micromanipulator. The entire brace is fixed to the lateral, rack-and-pinion coarse movement of a Huxley-type micromanipulator visible behind. Scale bar, 1 cm. (**C**) Halfcells of the Ag|AgCl-KCl type constructed (*left*) using 0.5 mm diameter Ag wire soldered to a 2-mm diameter socket threaded in a PTFE sleeve and fitted with silicon and glass tubing, and (*right*) using 0.5 mm diameter Ag wire soldered to a 2-mm diameter socket and press-fit with a silicon plug behind the tip segment of a 2-ml graduated polypropylene pipette tip. Scale bar, 1 cm. When backfilled with KCl electrolyte, the halfcells weigh 5.5 g (*left*) and 0.6 g (*right*). For general details of halfcell construction, see [9].

1. Pull microelectrodes to give tip resistances of 300–500 MΩ when filled with 200 mM K^+^-Ac.

2. For double-barrelled microelectrodes with the higher input resistances (and correspondingly lower electrolyte leakage rates), pull double-barrelled microelectrodes, after twisting 360^o^[9], using settings to give a pull time around 25 s. **NOTE**: *We use settings similar to those used for Vicia and tobacco guard cells*[34]*, but with the coil heat elevated to give pull times roughly 25% less than used for Vicia guard cells. The resulting microelectrodes have 1.8-2.0 cm-long shanks and tips that tapered with a 1–1.5*^*o*^*angle (Figure*2*A).*

3. Store microelectrodes in a glass desiccator and coat microelectrodes with paraffin before impalement for reducing capacitance [8,9,12].

### Key steps for impaling Arabidopsis guard cells

#### *Before starting*

Electrical recordings using double-barrelled microelectrodes are carried out largely as described previously [12,35] with some modifications. For K^+^ currents, microelectrode barrels are filled with 200 mM K^+^-Acetate, pH 7.5, to minimise interference from the anion current and recordings are carried out in continuously-flowing RB1; for measurements of anion current_,_ both electrode barrels are filled with 200 mM CsCl and the cells bathed in flowing RB2. Currents recorded under voltage clamp are normalised to the surface area of the impaled guard cells and, for K^+^ channel analysis, are corrected for background (instantaneous) currents as described previously [12,35] using Henry’s EP suite software (Y-Science, University of Glasgow, UK). **NOTE:***The typical length and radius of Arabidopsis guard cells are 20 and 5 μm, respectively. For the data summarised in the Tables, these parameters were 22 ± 0.6 μm*^*2*^*and 4 ± 0.1 μm, respectively. Assuming a spheroid geometry, the mean guard cell surface area and volume were 468 ± 12 μm*^*2*^*and 783 ± 21 μm*^*3*^*, respectively.*

An essential prerequisite is the use of a stable microelectrode mount that can accommodate two amplifier headstages and halfcells with a minimum of mechanical relaxation over time. We have adapted a Huxley-type micromanipulator with a custom-machined aluminium brace that supports positioning clamps (Narashige, C2-type) to stabilise paired amplifier headstages (Figure 2). Additionally, connections between the headstages and microelectrode barrels are made using Ag-AgCl|KCl halfcells similar to those described previously [9], but constructed around the light-weight polypropylene tubing from the tips of disposable 2-ml pipettes, which is essential to provide mechanical stability for long-term recordings (Figure 2).

1. Carry out impalement by first positioning the microelectrode to rest over one guard cell and present the tip across the stomatal pore before advancing along the axis of the microelectrode to impale the second guard cell. **NOTE**: *The initial movement of the microelectrode towards the guard cell requires very gentle manipulation.* A *‘snapping’ of the tip through the cell wall and into the guard cell should occur together with an increase in input resistance to approximately 1 GΩ and decrease (more negative) in membrane potential* (see Additional file 1: Table S1).

## Comments

### Buffer pretreatment and recording stability

Impalements are easier to achieve, and can be held for longer time when epidermal peels are pretreated with OB similar to that used by Allen et al. [36]. For comparison, the data in Tables 1, 2, 3, 4 and Additional file 1: Table S 1 summarise measurements from the guard cells of 407 stomata, including measurements of stomatal aperture, free-running membrane voltage, inward- and outward-rectifying K^+^ currents, I_K,in_ and I_K,out_, respectively, and in separate experiments of anion current, I_anion_. The data sets include measurements with and without OB pretreatment and show that stomata across all the lines tested were significantly more open (P < 0.05) following OB pretreatment: mean apertures following OB treatments were 3.22 ± 0.09 and 3.48 ± 0.09 μm at the start of measurements in RB1 and RB2, respectively, compared to 2.95 ± 0.04 and 2.89 ± 0.06 μm without OB pretreatment (see Table 1). Most important, the comparison shows that OB pretreatment greatly extends the time over which impalements can be held. Stable current recordings were extended by 62% and 83% for K^+^ and anion current studies, respectively – to periods often in excess of one hour – compared with experiments in which guard cells were impaled immediately after peeling and mounting (see Table 2). The capacity to extend electrical recordings over this time scale ensures that experimental challenges such as exposures to hormones and different environmental parameters (for example CO_2_, light, Ca^2+^ and other ion concentrations) can be carried out on a cell-by-cell basis in *Arabidopsis* guard cells much as was pioneered in guard cells of *Vicia* and tobacco [12,13,35,37,38]. In effect, work over these timescales enables the use of each cell as its own control. The following summaries are provided in conjunction with the tabulated data.

**Table 1:** **Effect of pretreatment with opening buffer (OB) on stomatal aperture in all Arabidopsis lines Col-0, *nia1nia2 *, *QC3, QL3, kc1-3, syp121, syp121ox, and dhar1-3 ***

	**Average of 8 lines**		***Col-0***		***nia1nia2 ***		***QC3***	
**Aperture (μm)**	**Control**	**Pretreatment**	**Control**	**Pretreatment**	**Control**	**Pretreatment**	**Control**	**Pretreatment**
I_K_ experiments	2.95±0.04 (150)	3.22±0.09* (99)	3.05±0.07 (79)	3.55±0.15** (39)	2.41±0.11 (12)	2.87±0.09* (58)	3.12±0.09 (46)	3.63±0.15** (15)
I_anion_ experiments	2.89±0.06 (84)	3.48±0.09** (74)	2.89±0.06 (28)	3.45±0.13** (30)	2.47±0.11 (23)	3.00±0.12** (25)	3.17±0.09 (33)	3.83±0.16** (19)

Data are means ± SE of (n) experiments.

* P < 0.05; **P < 0.01 as compared with control. *Arabidopsis* lines and mutants as indicated: the nitrate reductase null mutant *nia1-1/nia2-5* (*nia1/nia2*) [58,59] and ABA-receptor quadruple mutant *pyr1/pyl1/pyl2/pyl4* (*QC3 and QL3*) [60], the vesicle trafficking mutant *syp121* (=*syr1*/*pen1*) and the *syp121*ox over expression line [53], the dehydroascobate reductase mutant (*dhar1-3)* [62], and K^+^ channel mutant *kc1-2* [53]. All lines were in the *Arabidopsis* Columbia-0 (Col-0) background except QL3, which was in the Landsberg (Ler) background.

**NOTE***: Stomatal apertures were measured in epidermal peels of young leaves from three to five weeks old Arabidopsis plants. All operations were carried out on an Axiovert 135 fitted with Nomarski Differential Interference Contrast optics and an AxioCam digital camera system (Zeiss, Jena, Germany). All measurements were conducted in continuous flowing solutions. For measurements of apertures, 8–12 stomata were selected and their images recorded at 5-min intervals for subsequent analysis using Image J v.1.42(http://rsbweb.nih.gov/ij/). Apertures and dimensions of impaled guard cells were determined using a calibrated eyepiece micrometer and cell volumes calculated assuming a spheroid geometry.*

**Table 2:** **Effect of pre-treatment with opening buffer (OB) on seal lasting time of guard cells from all Arabidopsis lines Col-0, *nia1nia2 *, *QC3, QL3, kc1-3, syp121, syp121ox, and dhar1-3 ***

	**Average of 8 lines**		**Col-0**		***nia1nia2 ***		***QC3 ***	
**Time (min)**	**Control**	**Pretreatment**	**Control**	**Pretreatment**	**Control**	**Pretreatment**	**Control**	**Pretreatment**
I_K_ μA cm^-2^ experiments	21.2±4.0 (179)	34.4±3.8** (49)	19.2±1.5 (72)	36.8±3.1** (14)	18.6±2.6 (7)	33.7±2.7* (34)	28.8±3.6 (45)	40.0±6.8* (12)
I_anion_ μA cm^-2^ experiments	19.1±3.6 (45)	35.0±4.6** (29)	19.1±3.2 (12)	28.3±4.9* (9)	17.0±3.5 (15)	37.5±5.2** (12)	22.7±3.2 (13)	52.0±14.2** (5)

All the experiments lasted less than 10 min are discarded. Data are means ± SE of (n) experiments.

* P < 0.05; **P < 0.01 as compared with control.

**Table 3:** **Analysis of maximal conductance (g **_**max **_**), gating charge (δ), half maximal voltage (V **_**1/2 **_**) of I **_**K,in **_**and I **_**K,out **_**for the Arabidopsis lines Col-0, *nia1nia2 *, *QC3, QL3, kc1-3, syp121, syp121ox, and dhar1-3 *in both opening and non-condition **

**Channel**	**Parameters**	**Average of 8 lines**		**Col-0**		***nia1nia2 ***		***QC3 ***	
		**Control**	**Pretreatment**	**Control**	**Pretreatment**	**Control**	**Pretreatment**	**Control**	**Pretreatment**
I_K,in_	g_max_ (mS cm^-2^)	2.3±0.1 (103)	2.0±0.2 (67)	2.2±0.2 (26)	2.6±0.2* (15)	0.3±0.1 (10)	0.9±0.1** (36)	3.4±0.3 (21)	3.2±0.2 (13)
	δ	2.2±0.1 (103)	1.9±0.1* (67)	2.4±0.1 (26)	2.0±0.1* (15)	1.1±0.2 (10)	1.9±0.1** (36)	2.1±0.2 (21)	1.9±0.2 (13)
	V_1/2_ (mV)	-186±2.1 (103)	-182±1.6 (67)	-185±3 (26)	-182±2 (15)	-180±10 (10)	-179±2.5 (36)	-185±4.3 (21)	188±2.6 (13)
I_K,out_	g_max_ (mS cm^-2^)	3.0±0.1 (103)	3.5±0.1* (67)	2.6±0.2 (26)	3.8±0.6** (15)	2.7±0.3 (10)	3.6±0.2* (36)	3.9±0.3 (21)	3.5±0.3 (13)
	δ	1.6±0.1 (103)	1.6±0.1 (67)	1.8±0.1 (26)	1.6±0.1 (15)	1.5±0.1 (10)	1.5±0.1 (36)	1.5±0.2 (21)	1.5±0.2 (13)
	V_1/2_ (mV)	0.9±1.7 (103)	-3.6±1.7 (67)	6.0±2.6 (26)	0.4±2.3 (15)	-10±4.6 (10)	-2.4±2.4 (36)	-4.1±4.1 (21)	-6.5±5.7 (13)

Data are means ± SE of (n) experiments.

* P < 0.05; **P < 0.01 as compared with control.

**Table 4:** **Effect of pre-treatment with opening buffer (OB) on the ‘rundown’ of I **_**K,in **_**and I **_**K,out **_**in Arabidopsis guard cells of Col-0, *nia1nia2 *and *QC3 ***

**Channel**	**Current (μA cm**^**-2**^**)**	**Col-0**		***nia1nia2 ***		***QC3***	
		**10 min**	**30 min**	**10 min**	**30 min**	**10 min**	**30 min**
I_K,in_	Control	-484.1 ±185.6	-6.43 ±3.3**	-118.7±30.2	-1.2±10.3**	-413.8±142.1	-8.1±0.3**
	Pretreatment	-586.3 ±40.7	-431.9±75.2	-223.0 ±49.5	-247.1±54.8	-602.1±112.4	-543.2±44.3
I_K,out_	Control	598.2 ±89.0	145.29±36.0**	527.9±90.1	71.2±10.4**	591.2±111.1	168.4±119.5**
	Prereatment	901.0 ±271.5	921.64 ±236.4	698.8 ±147.2	669.9±174.9	741.3±198.8	732.3±127.0

Data are means ± SE from the analysis of >5 experiments extending over 60 min or more in each case.

**P < 0.01 as compared with control.

### K^+^ channel currents

**Figure 3:**
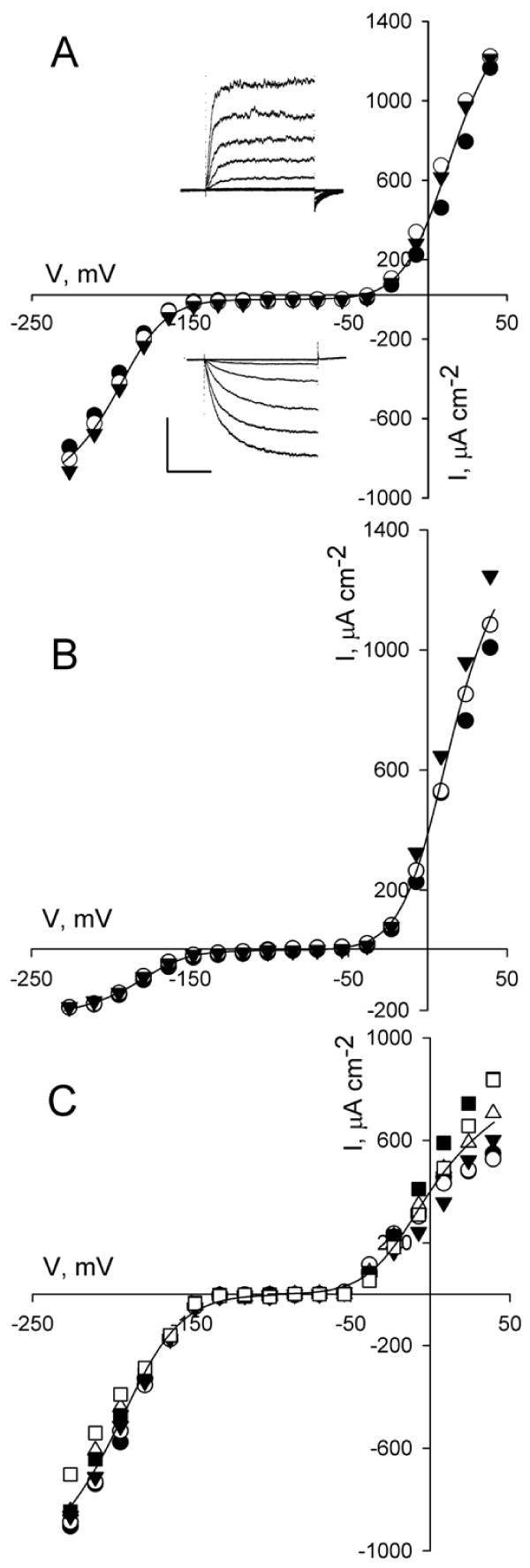
**I**_**K,in**_**and I**_**K,out**_**of wild-type (A), *nia1nia2 *(B), and *QC3 *(C) mutant *Arabidopsis *guard cells following pretreatment with opening buffer (OB).** (**A**) Steady-state current–voltage curves for I_K,in_ and I_K,out_ from one guard cell of wild-type *Arabidopsis *recorded at intervals over 30 min after 2-h OB pretreatment. Shown are data for voltage clamp scans taken at 10 (closed circles), 20 (open circles), and 30 min (closed triangles) after impalement. Clamp scans were from a holding voltage of −100 mV with tail steps to −100 mV. Test voltage steps were to voltages between −80 and +50 mV for I_K,out_ and to voltages between −100 and −240 mV for I_K,in_. Current–voltage curves were fitted jointly to a Boltzmann function (solid lines) and yielding values for g_max_ of 3.8 and 6.3 μS cm^-2^, V_1/2_, of −181 and +1 mV, and δ of 1.9 and 1.8 for I_K,in_ and I_K,out_, respectively. *Insets*: Current traces for time points at 30 min. Scale: 500 μA cm^-2^ vertical, 2 s horizontal. (**B**) Steady-state current–voltage curves for I_K,in_ and I_K,out_ from one guard cell of *nia1nia2* mutant *Arabidopsis* recorded at intervals over 30 min after 2-h OB pretreatment. Shown are data for voltage clamp scans taken at 10 (closed circles), 20 (open circles), and 30 min (closed triangles) after impalement. Clamp voltage scans as above. Current–voltage curves were fitted jointly to a Boltzmann function (solid lines) and yielding values for g_max_ of 0.9 and 6.1 μS cm^-2^, V_1/2_, of −178 and +5 mV, and δ, of 1.8 and 1.8 for I_K,in_ and I_K,out_, respectively. (**C**) Steady-state current–voltage curves for I_K,in_ and I_K,out_ from one guard cell of *nia1nia2 *mutant *Arabidopsis *recorded at intervals over 60 min after 2-h OB pretreatment. Shown are data for voltage clamp scans taken at 10 (closed circles), 20 (open circles), and 30 (closed triangles) 40 (open triangles), 50 (closed squares) and 60 (open squares) min after impalement. Clamp voltage scans as above. Current–voltage curves were fitted jointly to a Boltzmann function (solid lines) and yielding values for g_max_ of 4.1 and 4.6 μS cm^-2^, V_1/2_, -182 and −7 mV, and δ, of 1.7 and 1.9 for I_K,in_ and I_K,out_, respectively. **NOTE**: *Data analysis and curve fittings were carried out using SigmaPlot 11 (Systat Software, Inc., USA) and are reported, where appropriate, as means ± SE of n observations. Where appropriate significance was determined using Students’ T-test. Gating characteristics for I*_*K,in*_*and I*_*K,out*_*were determined by fitting steady-state current–voltage curves to Eqn.*(1)*using non-linear, least-squares minimisation and the Marquardt-Levenberg algorithm*[39].

**Figure 4:**
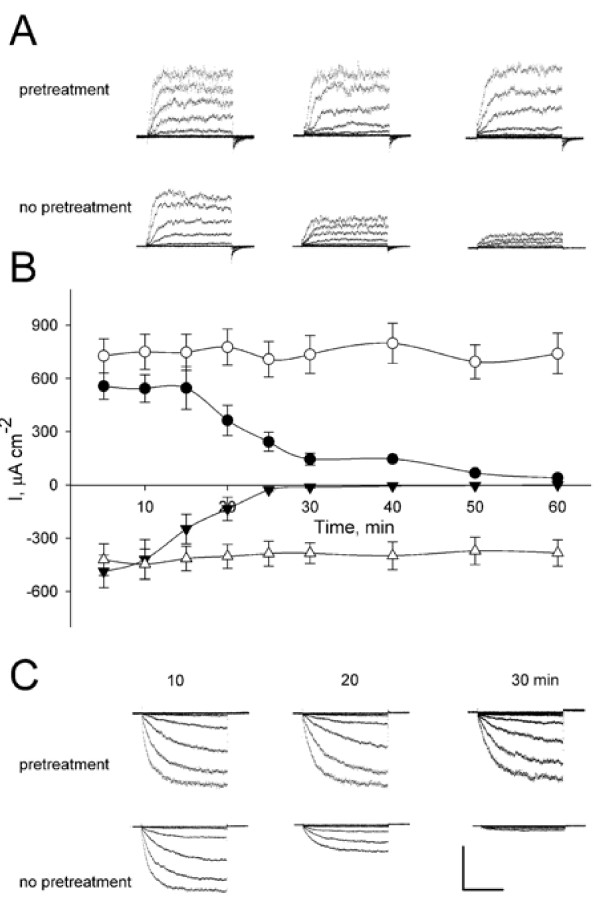
**Decay in I**_**K,in**_**and I**_**K,out**_**from guard cells of wild-type *Arabidopsis *plants without (no pretreatment) and with opening buffer pretreatment (pretreatment)Decay in I**_**K,in**_**and I**_**K,out**_**from guard cells of wild-type *Arabidopsis *plants without (no pretreatment) and with opening buffer pretreatment (pretreatment).** Voltage clamp scans were carried out at intervals following impalements. Raw current traces are shown in for scans at 10, 20, and 30 min time points from two guard cells for I_K,out_ (**A**) and I_K,in_ (**C**). Scale: vertical, 500 μA cm^-2^; horizontal, 2 s. Clamp scans were from a holding voltage of −100 mV with tail steps to −100 mV. Test voltage steps were to voltages between −80 and +50 mV for I_K,out_ and to voltages between −100 and −240 mV for I_K,in_. Data in (**B**) summarise the two current amplitude means ± SE (filled circles, no pretreatment; open circles, pretreatment) from 12 independent experiments with I_K,out_ determined at +40 mV and I_K,in_ determined at −220 mV. Note that currents recorded from guard cells in control experiments without OB pretreatment generally decayed with halftimes of 15–20 min.

Out of 275 independent experiments with measurements of the K^+^ currents 88% showed I_K,in_ activity and 100% yielded I_K,out_ activity as judged by the current activation kinetics, voltage dependencies and block by Cs^+^ and TEA^+^ (not shown, see Roelfsema and Prins [26,27], Forestier et al. [28] and Blatt et al. [38]). Guard cells pretreated with OB showed appreciably greater stability in both I_K,in_ and I_K,out_ over extended time periods compared with guard cells impaled without pretreatment (Figures 3, 4 and Tables 3 and 4). Mean I_K,in_ and I_K,out_ amplitudes of all of the lines tested at 30 min, for example, decayed to less than 2% and 22%, respectively, of the initial amplitudes recorded 10 min after impalements in guard cells without OB pretreatment (see also [26]). By contrast, the K^+^ currents showed less than a 5% change in amplitude over the same time period when guard cells were first pretreated in OB.

For quantitative comparisons, the steady-state kinetic characteristics for the K^+^ currents were fitted either individually or jointly to a Boltzmann function of the form

(1) $$I=\frac{g_{\text{max}}\left(V-E_{K}\right)}{(1+\text{exp}(-\delta \left(V_{1/2}-,V\right)F/RT)}$$

where E_K_ is the equilibrium voltage for K^+^ across the membrane, g_max_ is the maximum ensemble conductance for the channels, δ is the voltage sensitivity coefficient or gating charge and V_1/2_ is the voltage at which the ensemble conductance equals g_max_/2. Both approaches yielded parameter values that are statistically indistinguishable (Table 3) and are similar to those obtained previously for *Arabidopsis* as well as *Vicia* and tobacco guard cells [13,24,26-28,37,40].

Comparisons of the intrinsic gating characteristics for the different *Arabidopsis* lines and the overall means showed that OB pretreatment had no substantive effect on either δ or V_1/2_ (see also Figure 3). Values for g_max_ for I_K,out_ showed a significant increase in both the wild-type and *nia1nia2* mutant lines, whereas g_max_ was largely unaffected in the *QC3* mutant line (Table 3). These activities were reflected also in differences in the free-running membrane voltages (see Additional file 1: Table S 1). We note, too, a close similarity in the gating parameters δ and V_1/2_ between all of the lines, with the exception of the *syp121* and *nia1nia2* mutants for which the genetic deletions are expected to affect channel gating or K^+^ nutrition and balance [31]. Overall, these results confirm that the underlying gating properties for the two classes of K^+^ channels were unaffected, at least during the first hour after impalements.

### Anion current

**Figure 5:**
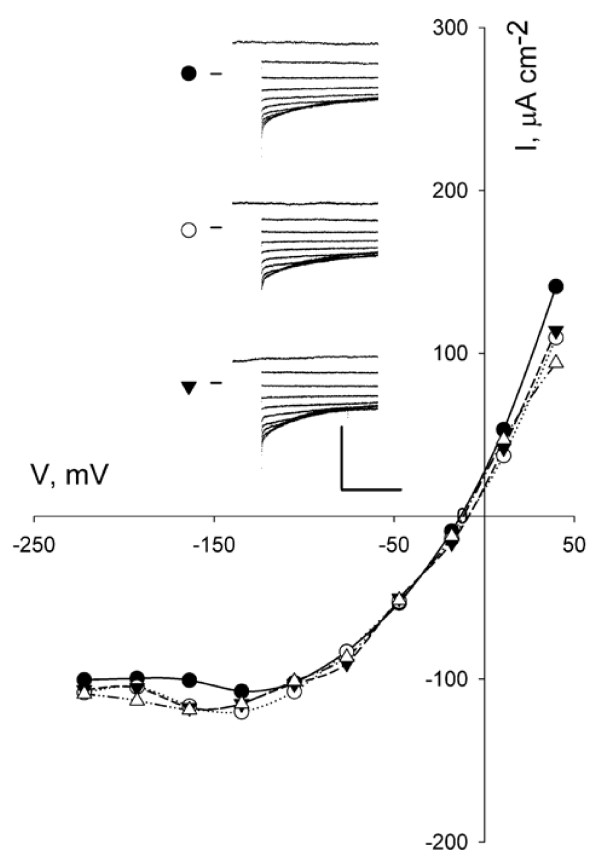
**Effect of pre-treatment with opening buffer (OB) on I **_**anion**_**in wild-type *Arabidopsis *.** Steady-state current–voltage curves for I_anion_ from one guard cell recorded after 2-h pretreatment with OB. Current–voltage curves for I_anion_ are not corrected for background. Rundown in this cell was evident only after 65 min. Data shown are taken from voltage clamp scans at 10 (closed circles), 20 (open circles), and 30 (inverted closed triangles) and 40 min (open triangles) after the impalement. Conditioning voltage was +50 mV with 10-s steps to voltages between +50 mV and −220 mV. *Inset:* Raw current traces for recordings at 10, 20 and 30 min cross-referenced by symbol. Scale: vertical, 300 μA cm^-2^; horizontal, 5 s.

To date, only Forestier et al. [28] reported I_anion_ in intact *Arabidopsis* stomatal guard cells, although components of I_anion_ have been identified with the *SLAC1* and *ALMT12* gene products [41-44]. We recorded I_anion_ in over 95% of cases from 158 guard cells in RB2 with current similar to past measurements from *Arabidopsis*, *Vicia* and tobacco [28,35,40]. The mean membrane voltage of −9.9 ± 1.6 mV in RB2 was also comparable to those recorded in these previous studies. We found no appreciable difference between guard cells with or without OB pretreatment (Additonal file 1: Table S 1) but, again, pretreatment prolonged the timeframe for I_anion_ recordings and experiments frequently extended over periods of one hour (Table 2 and Figure 5). Thus OB pretreatment improved the stability of I_anion_ recordings much as it did for those of I_K,in_ and I_K,out_.

## Summary

Three key factors are essential for successful, two-electrode, voltage clamp recordings with *Arabidopsis* guard cells. First, the preparation and handling of the plants is important, incorporating a pretreatment regime with a stomatal opening buffer prior to the start of experiments; second, microelectrode design must meet the demands for impalements of very small cells, notably in the use of fine tips with input resistances roughly 5-fold higher than typically used for *Vicia* and tobacco guard cells; finally, a modified clamp and brace to carry the amplifier headstages and construction of light-weight, but rigid halfcells are essential prerequisites to provide stability without mechanical relaxation for long-term recordings. Overall, this combination of factors is sufficient to achieve measurements comparable to those with the much larger guard cells of *Vicia* and tobacco. These methods should now greatly speed the analysis of many mutants of *Arabidopsis* by simplifying electrophysiological studies of the guard cells.

## Authors’ contributions

ZHC carried out the electrophysiological studies and analysed the data together with XQX; CE carried out aperture measurements and image analysis; AH and MRB developed the software utilities, mechanical and electrical hardware for the voltage clamp recordings; ZHC, CE and MRB wrote the manuscript. All authors read and approved the final manuscript.

## Supplementary Material

Additional file 1 **Table S1.** Effect of pre-treatment with opening buffer (OB) on guard cell membrane potential (E_m_) in all Arabidopsis lines Col-0, *nia1nia2*, *QC3, QL3, kc1-3, syp121, syp121ox, and dhar1-3*. Data are means ±SE of (n) experiments. Click here for file
