# Supplementary material for: Protocol: optimised electrophyiological analysis of intact guard cells from Arabidopsis
Source: Plant Methods. 2012 May 6;8:15. doi: 10.1186/1746-4811-8-15 (PMC3475070; doi:10.1186/1746-4811-8-15)
Supplement: Additional file 1 — Table S1. Effect of pre-treatment with opening buffer (OB) on guard cell membrane potential (Em) in all Arabidopsis lines Col-0, nia1nia2, QC3, QL3, kc1-3, syp121, syp121ox, and dhar1-3. Data are means ±SE of (n) experiments. [file 1746-4811-8-15-S1.doc]

**Table S1.** Effect of pre-treatment with opening buffer (OB) on guard cell membrane potential (E_m_) in all Arabidopsis lines Col-0, *nia1nia2*, *QC3, QL3, kc1-3, syp121, syp121ox, and dhar1-3*. Data are means ±SE of (n) experiments.

|  | Averages of 8 lines | | Col-0 | | *nia1nia2* | | *QC3* | |
| --- | --- | --- | --- | --- | --- | --- | --- | --- |
| E_m_ (mV) | Control | Pretreatment | Control | Pretreatment | Control | Pretreatment | Control | Pretreatment |
| I_K_ experiments | −49.8±1.0 (207) | −70.1±1.6** (181) | −49.9±2.1 (102) | −68.8±2.2** (72) | −46.0±0.9 (16) | −61.6±2.7* (60) | −61.6±1.9 (69) | −76.2±1.2**  (16) |
| I_anion_ experiments | −5.6±2.2  (74) | −11.2±2.2  (63) | −9.6±4.0  (25) | −12.1±3.0  (33) | −13.9±4.4 (19) | −19.5±3.6  (22) | 0.3±3.0  (32) | −7.5±5.1  (18) |

*, P<0.05; **P<0.01 as compared with control
